# Circular RNA *circRNF13* inhibits proliferation and metastasis of nasopharyngeal carcinoma via SUMO2

**DOI:** 10.1186/s12943-021-01409-4

**Published:** 2021-08-31

**Authors:** Yongzhen Mo, Yumin Wang, Shuai Zhang, Fang Xiong, Qijia Yan, Xianjie Jiang, Xiangying Deng, Yian Wang, Chunmei Fan, Le Tang, Shanshan Zhang, Zhaojian Gong, Fuyan Wang, Qianjin Liao, Can Guo, Yong Li, Xiaoling Li, Guiyuan Li, Zhaoyang Zeng, Wei Xiong

**Affiliations:** 1 NHC Key Laboratory of Carcinogenesis and Hunan Key Laboratory of Cancer Metabolism, Hunan Cancer Hospital and Affiliated Cancer Hospital of Xiangya School of Medicine, Central South University, Changsha, 410078 Hunan China; 2 Key Laboratory of Carcinogenesis and Cancer Invasion of the Chinese Ministry of Education, Cancer Research Institute, Central South University, Changsha, 410078 Hunan China; 3 Department of Otolaryngology Head and Neck Surgery, Xiangya Hospital, Central South University, Changsha, 410078 Hunan China; 4 Department of Stomatology, Xiangya Hospital, Central South University, Changsha, 410078 Hunan China; 5 Department of Oral and Maxillofacial Surgery, The Second Xiangya Hospital, Central South University, Changsha, 410011 Hunan China; 6 Department of Medicine, Dan L Duncan Comprehensive Cancer Center, Baylor College of Medicine, Houston, TX USA

**Keywords:** Nasopharyngeal carcinoma, *circRNF13*, SUMO2, GLUT1, Glycolysis, Proliferation, Metastasis

## Abstract

**Background:**

Circular RNAs (circRNAs) are widely expressed in human cells and are closely associated with cancer development. However, they have rarely been investigated in the context of nasopharyngeal carcinoma (NPC).

**Methods:**

We screened a new circRNA, *circRNF13*, in NPC cells using next-generation sequencing of mRNA. Reverse transcription polymerase chain reaction and RNA fluorescence in situ hybridization were used to detect *circRNF13* expression in 12 non-tumor nasopharyngeal epithelial (NPE) tissues and 36 NPC samples. Cell proliferation was detected using MTT and flow cytometry assays, and colony formation capability was detected using colony formation assays. Cell migration and invasion were analyzed using wound-healing and Transwell assays, respectively. Cell glycolysis was analyzed using the Seahorse glycolytic stress test. Glucose transporter type 1 (GLUT1) ubiquitination and SUMOylation modifications were analyzed using co-immunoprecipitation and western blotting. *CircRNF13* and Small Ubiquitin-like Modifier 2 (SUMO2) interactions were analyzed using RNA pull-down and luciferase reporter assays. Finally, to test whether *circRNF13* inhibited NPC proliferation and metastasis in vivo, we used a xenograft nude mouse model generated by means of subcutaneous or tail vein injection.

**Results:**

We found that *circRNF13* was stably expressed at low levels in NPC clinical tissues and NPC cells. In vitro and in vivo experiments showed that *circRNF13* inhibited NPC proliferation and metastasis. Moreover, *circRNF13* activated the SUMO2 protein by binding to the 3′- Untranslated Region (3′-UTR) of the SUMO2 gene and prolonging the half-life of SUMO2 mRNA. Upregulation of SUMO2 promotes GLUT1 degradation through SUMOylation and ubiquitination of GLUT1, which regulates the AMPK-mTOR pathway by inhibiting glycolysis, ultimately resulting in the proliferation and metastasis of NPC.

**Conclusions:**

Our results revealed that a novel *circRNF13* plays an important role in the development of NPC through the *circRNF13*-SUMO2-GLUT1 axis. This study implies that *circRNF13* mediates glycolysis in NPC by binding to SUMO2 and provides an important theoretical basis for further elucidating the pathogenesis of NPC and targeted therapy.

**Supplementary Information:**

The online version contains supplementary material available at 10.1186/s12943-021-01409-4.

## Background

Nasopharyngeal carcinoma (NPC) is a malignant tumor of epithelial origin in the nasopharynx, with an incidence rate of up to 2 in 1,000 in Southern China and Southeast Asia [1–4]. Because NPC occurs in the nasopharynx, its early symptoms are not obvious, but NPC have a strong tendency to invade and metastasize. Clinical studies show that approximately 70% of patients with NPC experience metastasis to the lymph nodes in the neck, and almost all patients in advanced stages have invasive growth to the skull base [5–7]. However, to date, the molecular mechanisms involved in the development of NPC, especially in the metastatic process, are still unclear. Therefore, further understanding of the pathogenesis of NPC is needed to develop more effective therapeutic strategies to combat this disease.

Circular RNAs (circRNAs) are a newly discovered class of noncoding RNAs in recent years, and the study of their biological function and relationship with diseases has become a frontier of biomedical research [8–11]. CircRNAs are a group of RNAs with a loop-like structure that lacks a polyA tail. They are not easily degraded by RNA enzymes and have very stable properties due to their special loop structure [12–15]. With the development of the next-generation sequencing technology in recent years, an increasing number of circRNAs have been discovered, and more than 30,000 circRNAs have currently been identified in the transcriptome of various human cell types [16, 17]. An increasing number of studies have found that circRNAs have important functions in numerous life processes and are involved in the development of many human diseases, including malignant tumors [18, 19]. Although many circRNAs have been identified, the functions of the vast majority of circRNAs are still unclear, especially in NPC.

Intracellular protein ubiquitination is an important post-translational modification that is widely found in eukaryotic cells. The vast majority of intracellular proteins are degraded via the ubiquitin-dependent proteasome pathway. Small ubiquitin-like modifier 2 (SUMO2) is a member of the SUMO family, and SUMOylation is a type of ubiquitin-like protein modification. SUMOylation and ubiquitination often occur at the same lysine residues of a substrate protein, and SUMOylation can sometimes antagonize ubiquitination in the regulation of transcription factors [20]. SUMOylation can also promote the ubiquitination and degradation of modified cytoplasmic, mitochondrial, and membrane proteins, serving as a signal for the recruitment of ubiquitin E3 ligases in various biological processes [21].

Glucose transporter proteins (GLUTs) are one of the most important transmembrane proteins in the body that are responsible for glucose transport and reabsorption in different tissues and organs of the body. High expression of GLUT1 in various tumors, such as liver, gastric, and breast cancers, is involved in the regulation of malignant phenotypes, such as tumor metastasis and proliferation [22, 23]. It provides favorable conditions for glycolysis through massive glucose uptake, providing more energy and synthetic raw materials for tumor cells. In addition, the large amount of lactic acid produced by glycolysis alters the microenvironment of tumor cells, which is more favorable for tumor cell invasion and metastasis.

In this study, we identified a novel circRNA molecule, *circRNF13* (circBase ID: has_circ_0001346), which is expressed at low levels in NPC. The proliferation and metastasis of *circRNF13* were assessed in NPC cells through in vitro and in vivo experiments. Further studies revealed that *circRNF13* directly binds and stabilizes SUMO2 mRNA, which upregulates the protein levels of SUMO2 and promotes GLUT1 degradation by means of SUMOylation and ubiquitination, thus inhibiting the glycolytic process and proliferation, migration, and invasion of NPC.

## Methods

### NPC clinical samples

In total, 12 non-tumor nasopharyngeal epithelial (NPE) tissues and 36 NPC samples were collected at the Affiliated Cancer Hospital of Central South University (Changsha, China). This study was approved by the Joint Ethics Committee of the Central South University Health Authority, and informed consent was obtained from all participants. Diagnoses of all specimens were confirmed via histopathological examination.

### Cell culture, plasmids, and transfection

NPC cell lines 5-8F, HNE2, CNE2, HONE1, and 6-10B and immortalized normal nasopharyngeal epithelial NP69 cells were obtained from the Cell Center of Central South University. All cells were cultured in RPMI-1640 medium (Gibco) supplemented with 10% fetal bovine serum (Gibco). All cells were maintained at 37 °C in a humidified incubator with 5% CO_2_.

The circRNA expression plasmid pCirc was generously provided by Prof. Li Yong at Baylor College of Medicine for RNA circulation. For *circRNF13* overexpression, cDNAs reverse-transcribed from RNAs of CNE2 cells were used as PCR templates and subjected to amplification of the full-length *circRNF13*. The reporter plasmid pMIR-Luciferase-Reporter for SUMO2 and inserts of the SUMO2 3’UTR sequence within the pMIR-Luciferase-Reporter plasmid were constructed with the One Step Cloning Kit (Vazyme). Plasmids were transfected into cells using Lipofectamine 3000 Reagent (Invitrogen, USA). Primers used are listed in Table S1.

### RNA isolation and RT-PCR

Total RNA was extracted using TRIzol reagent (Life Technologies) and subjected to reverse transcription with random primers using the 5 × All-In-One kit (Abm). Then, the expression levels of target RNAs were measured with SYBR Green Master Mix using a StepOnePlus Real-Time PCR System (Applied Biosystems). *β-actin* was used as an endogenous control, and the fold change was calculated via the 2^−∆∆CT^ method. Primers used are listed in Table S1.

### Cytosolic/nuclear fraction assay

Cells were resuspended in hypotonic buffer (25 mM Tris–HCl, pH 7.4, 1 mM MgCl_2_, 5 mM KCl) and incubated on ice for 5 min before adding an equal volume of hypotonic buffer containing 1% NP-40 for an additional 5 min. After centrifuging the cells at 5,000 g for 5 min, the supernatant was collected as the cytosolic fraction. Pellets were washed twice with hypotonic buffer and then resuspended in nuclear resuspension buffer (20 mM HEPES, pH 7.9, 400 mM NaCl, 1 mM EDTA, 1 mM EGTA, 1 mM DTT, 1 mM PMSF). After incubation on ice for 30 min, samples were centrifuged at 12,000 g for 10 min, and supernatants were collected as the nuclear fraction. Reverse transcription of RNA was used for RT-PCR detection. Primers used are listed in Table S1.

### RNase R treatment

RNase R treatment experiments were performed to verify the stability of circRNA. Five micrograms of total RNA was extracted from NPC cells and divided into two groups. One group was incubated with 1 μl RNase R (20 U/μl; Epicenter, WI, USA) at 37 °C for 30 min, and the other group was treated without RNase R. RNA was incubated at 70 °C for 10 min to inactivate RNase R and then reverse-transcribed for RT-PCR detection. Primers used are listed in Table S1.

### Fluorescence in situ hybridization (FISH)

A digoxigenin-labeled specific probe for *circRNF13* was designed and synthesized by Sangon. The FISH experiment was performed according to the manufacturer's instructions (GenePharma). First, cells were fixed and permeabilized using 0.25% Triton X-100. Then, cells were hybridized with specific probes overnight at 37 °C with *circRNF13* in hybridization buffer. Nuclei were counterstained with DAPI (Invitrogen, D1306, USA). Cells were imaged using a confocal microscope (Ultra-View Vox, Perkin-Elmer, Waltham, MA, USA). The *circRNF13* probe used is listed in Table S1.

### MTT and cell cycle assays

For MTT assays, 1,000 cells per well were seeded into 96-well plates for the MTT assay. Cells were incubated with 0.5 mg/mL filtered sterile MTT (Beyotime) at 37 °C for 4 h at the indicated time point. Then, the media were removed and replaced with 200 μL DMSO, and absorbance was measured at 490 nm.

Cell cycle assays were performed using flow cytometry according to the manufacturer's guidelines. To examine the cell cycle, measurements were performed. Briefly, cells were trypsinized, collected and stained in solution with RNase or PI for 15 min at 25 °C. Flow cytometry analysis was immediately performed using a FACSCalibur.

### Colony formation assay

For colony formation assay. In brief, 2,000 cells were plated into 6-well plates after transfected *circRNF13* overexpression vector or *circRNF13* siRNA for 48 h. The cells were allowed to grow for the next 7 days to allow colony formation and the colonies were visualized using crystal violet staining.

### Wound healing assay

Cells were cultured in 6-well plates for 24 h and then wounded using a sterilized pipet tip to make a straight scratch. After gentle washing with D-Hanks, cells were cultured in RPMI-1640 medium with 1% FBS. Pictures were taken using an Olympus digital camera at 0 and 24 h after wounding (at least three randomly selected fields were imaged).

### Transwell assays

Cell invasion assays were performed using a Transwell chamber (Millipore). RPMI-1640 medium supplemented with 20% FBS was added to the bottom chambers, and then cells were suspended in RPMI-1640 medium and seeded into the top chamber, which was coated with Matrigel. After incubating at 37 °C for 48 h, cells that did not migrate through the pores were removed using a cotton swab. The Transwell chambers were fixed in 4% paraformaldehyde for 30 min, followed by staining with 1% crystal violet for 10 min. Cells on the bottom of the chamber were counted using an inverted phase-contrast microscope (at least three randomly selected fields were quantified).

### Animal experiments

We purchased 4-week-old female BAL B/c nude mice from the Experimental Animal Center of Central South University (Changsha, China) and raised them in an SPF-free barrier environment. For lung metastasis experiments, nude mice were randomly divided into four groups (*n* = 7 per group). Each nude mouse was injected via the tail vein with 2 × 10^6^ NPC CNE2 cells transfected with the *circRNF13* overexpression vector, *circRNF13* siRNA, the blank plasmid, or scrambled siRNA. After eight weeks, nude mice were sacrificed by cervical dislocation. Lung tissue was removed, weighed, and imaged, and the number of nodules on the surface of the lung was recorded to assess tumor metastasis. Lung tissues were then subjected to gradient dehydration, sectioned, embedded in paraffin, and stained with H&E for histological examination.

For subcutaneous tumorigenesis experiments, nude mice were randomly divided into four groups (*n* = 7). Each nude mouse was subcutaneously injected with 3 × 10^6^ NPC CNE2 cells transfected with the *circRNF13* overexpression vector, *circRNF13* siRNA, or the blank plasmid, scrambled siRNA. Tumor growth was monitored every 3 days. Tumor size was assessed by measuring the largest perpendicular diameters, and tumor volume was calculated as follows: V = 1/2 × (length) × (width) × (width). Twenty-one days after subcutaneous inoculation, mice were euthanized by cervical dislocation, and the tumor tissue was excised. The formed tumor masses were removed and weighed. All animal protocols were approved by the Institutional Laboratory of Animal Care and Use Committee at Central South University.

### Hematoxylin–Eosin staining (H&E)

Paraffin mouse tissue sections were first heated at 65 °C for 2 h. After paraffin sections were dewaxed and hydrated, nuclei were stained with hematoxylin solution (Biosharp, Anhui, China), and then cytoplasmic staining was performed with eosin staining solution (Biosharp, Anhui, China). After the slices were dried, the sheets were preserved with neutral resin (SCR, Shanghai, China).

### Seahorse assays

Assays were performed using the Seahorse XFp analyzer (Seahorse Bioscience, Agilent) according to the manufacturer’s instructions. Briefly, 8000 cells/well were seeded into an 8-well XF cell culture microplate in growth medium 24 h before the assay. The extracellular acidification rate (ECAR) was measured using an XFp analyzer in XF base medium (pH = 7.4) containing 1 mM glutamine following sequential additions of glucose (10 mM), oligomycin (1.5 μM) and 2-DG (50 mM). Data were analyzed by the Seahorse XF Glycolysis Stress Test Report Generator package.

### Liquid chromatography-mass spectrometry (LC–MS/MS)

Mass spectrometry assays according to the manufacturer’s protocol with minor modifications. Briefly, Scrambled siRNA or *circRNF13* siRNA was transfected into CNE2 cells for 48 h. Total protein was extracted and digested with protease overnight. The digested peptide mixture was dried and treated with 0.1% trifluoroacetic acid (TFA). After diluting the 5 μL samples, we used an LTQ Orbitrap Velos Pro mass spectrometer (Thermo Scientific, Bremen, Germany) coupled with an Ultimate 3000 RSLC Nano System (Dionex, CA, USA) to recover proteins and perform proteomic analysis of total proteins, which were identified using Proteome Discoverer 1.4 software (Thermo Fisher Scientific, MA, USA), and the resulting original file was imported into the UniProt KB/Swiss-Prot database for searching. For the database search, the mass tolerances of the precursor and fragmentation were set to 10 ppm and 0.8 Da, respectively. Peptides with a false discovery rate < 1% (q value < 0.01) and proteins with an area value lower than 1 × 10^6^ were discarded. Proteins that met the following criteria were considered to be differentially expressed proteins: ≥ 2 peptides and ≥ 95% confidence; and an average fold change in protein levels ≥ 2.00 or ≤ 0.50. (Student's t-test, *p* < 0.05)**.**

### Enrichment analysis

The differentially expressed proteins screened by LC–MS/MS were imported to Ingenuity Pathway Analysis (IPA) software, and canonical pathways of differentially expressed genes were analyzed to obtain enrichment pathways. Fisher's exact test was used to calculate a *p*-value to determine the probability of each biological function.

### RNA pull-down

The biotin-labeled *circRNF13* probe was synthesized by Sangon Biotech, and the RNA pull-down assay was performed as previously described with minor modifications. *CircRNF13*-overexpressing HNE2 and CNE2 cells were fixed in 1% formaldehyde for 10 min, lysed, and sonicated. After centrifugation, 50 μL of the supernatant was retained as input, and the remaining part was incubated with a *circRNF13*-specific probe streptavidin Dynabeads (M-280; Invitrogen) mixture overnight at 4 °C. The next day, an M-280 Dynabeads probe-circRNA mixture was washed and incubated with 200 uL of lysis buffer. Finally, TRIzol was added to the mixture for RNA extraction and detection. The *circRNF13* probe and primers used are listed in Table S1.

### Dual-luciferase reporter assay

The Dual-Luciferase® Reporter Assay System was used according to the manufacturer’s instructions (Promega). To evaluate the interaction between *circRNF13* and SUMO2, *circRNF13* and pMIR-Luciferase-Reporter-SUMO23’UTR plasmids were transfected in HNE2 and CNE2 cells. Forty-eight hours later, firefly and Renilla luciferase activity was examined by the Dual-Luciferase Reporter Assay System, and Renilla activity was used to normalize firefly activity.

### In situ hybridization (ISH)

The ISH kit was purchased from Boster Biological (CA, USA). Paraffin mouse tissue sections were deparaffinized and rehydrated with gradient alcohol-water solution, and endogenous peroxidase was inactivated with 3% H_2_O_2_. An appropriate amount of pepsin (1 mL 3% citric acid solution and two drops of concentrated pepsin) was added to tissue specimens and digested at 37 °C for 15 min. The digestion was then quickly terminated with 0.1 mol/L glycine solution. After refixation with 4% paraformaldehyde, the prehybridization solution was used at 37 °C for 30 min, and hybridization was conducted using a digoxin-labeled *circRNF13* probe (Sangon, Biotech, Shanghai, China) overnight at 37 °C. The next day, slides were washed in 2 × SSC, 0.5 × SSC, and 0.2 × SSC. Biotinylated rat anti-digoxigenin, streptavidin–biotin complex (sABC), and biotinylated peroxidase were then added dropwise. After incubation for 30 min, slides were washed with PBS. DAB color developing solution was subsequently added for 5–10 min and then placed in running water to stop the color reaction. Sections were subjected to dehydration using gradient alcohol, and a neutral resin mount was added dropwise. The *circRNF13* probe used is listed in Table S1.

### Protein half-life assay

Initially, cells were transfected with the *circRNF13* overexpression vector or *circRNF13* siRNA for 48 h. Then, cycloheximide (CHX, 50 μg/mL) was added into cell culture medium at indicated time points.

### Immunoprecipitation

For immunoprecipitation, the antibodies were incubated with 35 μL of protein A/G magnetic beads (Bimake, Houston, Texas, USA) with constant rotation at room temperature for 2 h. HNE2 or CNE2 cell lysates were extracted using GLB^+^ lysis buffer (150 mM NaCl, 10 mM Tris–HCl pH 7.5, 0.5% Triton X-100, 10 mM EDTA pH 8.0) with a protease inhibitor cocktail (Roche, Basel, Switzerland, USA) on ice for 2 h. Lysates were centrifuged and then incubated with antibody-conjugated beads for 4 °C overnight. Next the antibody-bead complexes were washed 5–6 times with cold GLB^+^ lysis buffer. Then the precipitated proteins were resuspended and boiled 10 min using 6 × SDS-PAGE loading buffer. The boiled immune complex was put on ice for 2 min and subjected for SDS-PAGE electrophoresis. The primary antibodies used are listed in Table S2.

### Immunofluorescence

The cultured HNE2 and CNE2 cells were incubated with 4% paraformaldehyde and then blocked with 5% BSA. The cells were treated with specific antibodies at 4 °C overnight and the secondary antibodies at 37 °C for 1 h. And the cells were counterstained with DAPI for 10 min and imaged under a confocal microscope (Ultra-View Vox, Perkin-Elmer, Waltham, MA, USA). The primary antibodies used are listed in Table S2.

### Western blotting

Total protein lysate (40 μg per lane) was loaded on 10–15% Bis–tris polyacrylamide mini gels (Invitrogen). SDS-PAGE was run at 120 V for 1.5 h to 2 h. Proteins were transferred to nitrocellulose or PVDF membranes by wet transfer for 60–90 min at 100 V. Membranes were blocked in 5% nonfat dry milk in Tris-buffered saline supplemented with Tween 20 (0.1%) (TBST) or phosphate-buffered saline (PBS) for 60 min at room temperature. After blocking, membranes were cut horizontally to examine multiple proteins of different sizes on each gel. Membranes were incubated on a plate shaker overnight at 4 °C with primary antibodies diluted in TBS-T. Membranes were extensively washed with TBS-T (minimum 3 × for 10 min), followed by incubation with appropriate horseradish peroxidase-conjugated secondary antibodies diluted in TBS-T with 5% nonfat dry milk for 60 min at RT on a plate shaker. Membranes were extensively washed with TBS-T (minimum 4× for 15 min). Signals were detected using a Luminata Crescendo detection system following the manufacturer’s recommendations. Multiple film (HyBlot CL, Denville) exposures ranging from 2 s to 2 min were performed for optimal image analysis. The antibodies are listed in Table S2.

### Immunohistochemistry

Immunohistochemistry was performed on formalin-fixed paraffin-embedded sections of mouse xenograft tissues. Briefly, tissues were deparaffinized and rehydrated, and samples were subjected to EDTA-mediated high-temperature antigen retrieval; the samples were then incubated overnight at 4 °C with the primary antibodies. The staining was scored according to the staining intensity and the distribution of stained cells. The distribution was evaluated as none (0), ≤ 10% (1), 10%—50% (2), 50%—80% (3), and > 80% (4). Intensity was evaluated as none (0), faint (1), moderate (2), strong (3), or very strong (4). The sections were reviewed by two pathologists. The antibodies are listed in Table S2.

### Statistical analysis

Statistical analysis was performed using GraphPad Prism 7 software. Student’s t-tests were used to evaluate significant differences between any two groups of data, and one-way ANOVA was used to evaluate significant differences for multiple comparisons. All data are represented as mean ± Standard Deviation (SD). Differences were considered significant at *p* < 0.05. *, *p* < 0.05; **, *p* < 0.01; ***, *p* < 0.001.

## Results

### *CircRNF13* exhibits low expression in NPC clinical tissues

**Fig. 1:**
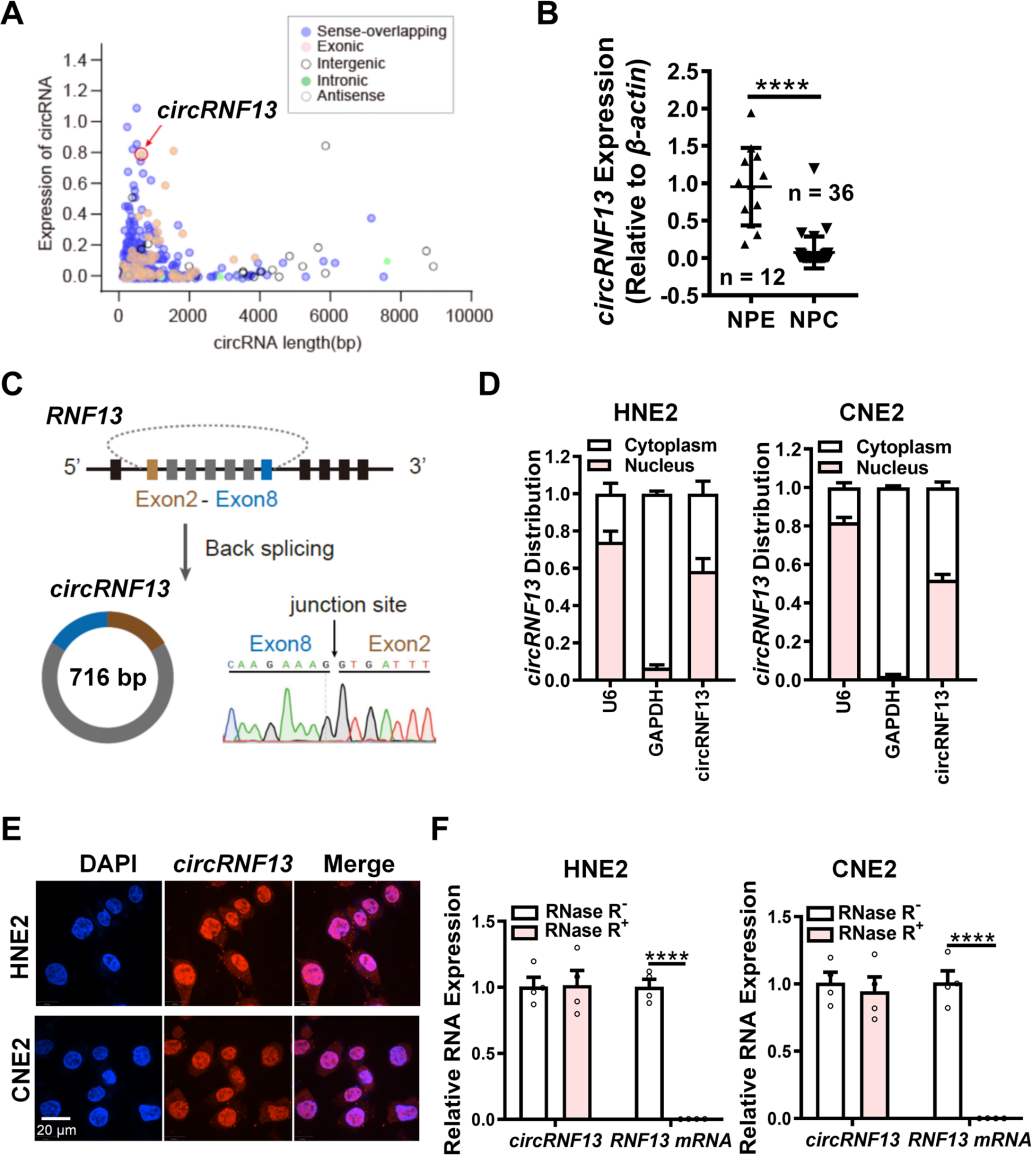
*CircRNF13* expression is lowly expressed in NPC. **A** Expression profile of circular RNAs (circRNAs) in 5-8F cells. Five types were included: exon-derived circRNAs, intron-derived circRNAs, anti-sense strand circRNAs, overlapping regions of sense strand circRNAs, and intergenic circRNAs, according to their different looping regions. *CircRNF13* was ranked 14th among the top 20, and was screened due to its high abundance (RPM = 0.59). **B** Expression of *circRNF13* was examined in NPC (*n* = 36) and NPE (*n* = 12) tissues using RT-PCR, NPE, non-tumor nasopharyngeal epithelial tissues. Data have been represented as mean ± standard deviation (SD). ****, *p* < 0.0001. **C** Schematic structure of *circRNF13*. *circRNF13* is 716 bp in length and circularly spliced by 2–8 exons of the *RNF13* gene (RefSeq: NM_183383.2) on the chromosome 3q25.1 region, as verified using Sanger sequencing. **D ***circRNF13* is primarily localized in the cytoplasm, as identified using nucleoplasmic separation experiments. *U6* was selected as a nuclear marker and *GAPDH* was selected as a cytoplasmic marker. **E** Intracellular localization of *circRNF13* was examined using fluorescence in situ hybridization. Scale bar: 20 μm. **F** Stability of *circRNF13* and RNF13 mRNA was detected in RNase R-treated HNE2 and CNE2 cells using RT-PCR. Data have been represented as mean ± SD. ****, *p* < 0.0001

To obtain the expression profiles of circRNAs in NPC, next-generation sequencing of mRNA (RNA-seq) data in the NPC cell line 5-8F (accession number: PRJNA391554) was reanalyzed [20]. A total of 6,153 distinct circRNAs were identified after excluding very low abundance circRNAs (average reads per million fragments mapped < 0.1). Among these circRNAs, five types were included: exon-derived circRNAs, intron-derived circRNAs, anti-sense strand circRNAs, overlapping regions of sense strand circRNAs, and intergenic circRNAs, according to their different looping regions (Fig. 1A). The expression of the top 20 circRNAs (Table S3), based on their abundance were assessed in 36 NPC tissues and 12 normal nasopharyngeal mucosal/epithelial (NPE) tissues (Table S4), using RT-PCR. Among them, *circMAN1A2*, *circFAM129B*, *circCAMSAP1*, *circCYDL*, *circARARB1*, *circPVT1*, and *circSETD3* were highly expressed in the 36 NPC tissues, as compared to the 12 NPE tissues (Fig. S1A). A novel potential circRNA, *circRNF13*, was expressed in high abundance in NPC tissues, but *circRNF13* expression was higher in NPE tissues than in NPC tissues (Fig. 1B). Further experiments showed that *circRNF13* was also expressed at lower levels in NPC cell lines (HNE2, CNE2, 5-8F, HONE1, and 6-10B) than in the normal NPE cell line NP69 (Fig. S1B). Based on the design of reverse primers for RT-PCR at the *circRNF13* splice site and Sanger sequencing of the RT-PCR products, we concluded that *circRNF13* is formed by circular splicing of the exons 2–8 of RNF13 gene (NM_183381.2) on the chromosome 3q25.1, with a full length of 716 bp (Fig. 1C). Nucleoplasmic separation assays and RNA fluorescence in situ hybridization assays showed that circRNF13 was distributed in both the nucleus and cytoplasm and was more localized in the nucleus. (Fig. 1D-E), while RNase R treatment revealed that *circRNF13* was more stable than RNF13 mRNA (Fig. 1F) in NPC cells. These data suggested that *circRNF13* may affect NPC development.

### *CircRNF13* inhibits proliferation, migration, and invasion of NPC cells

**Fig. 2:**
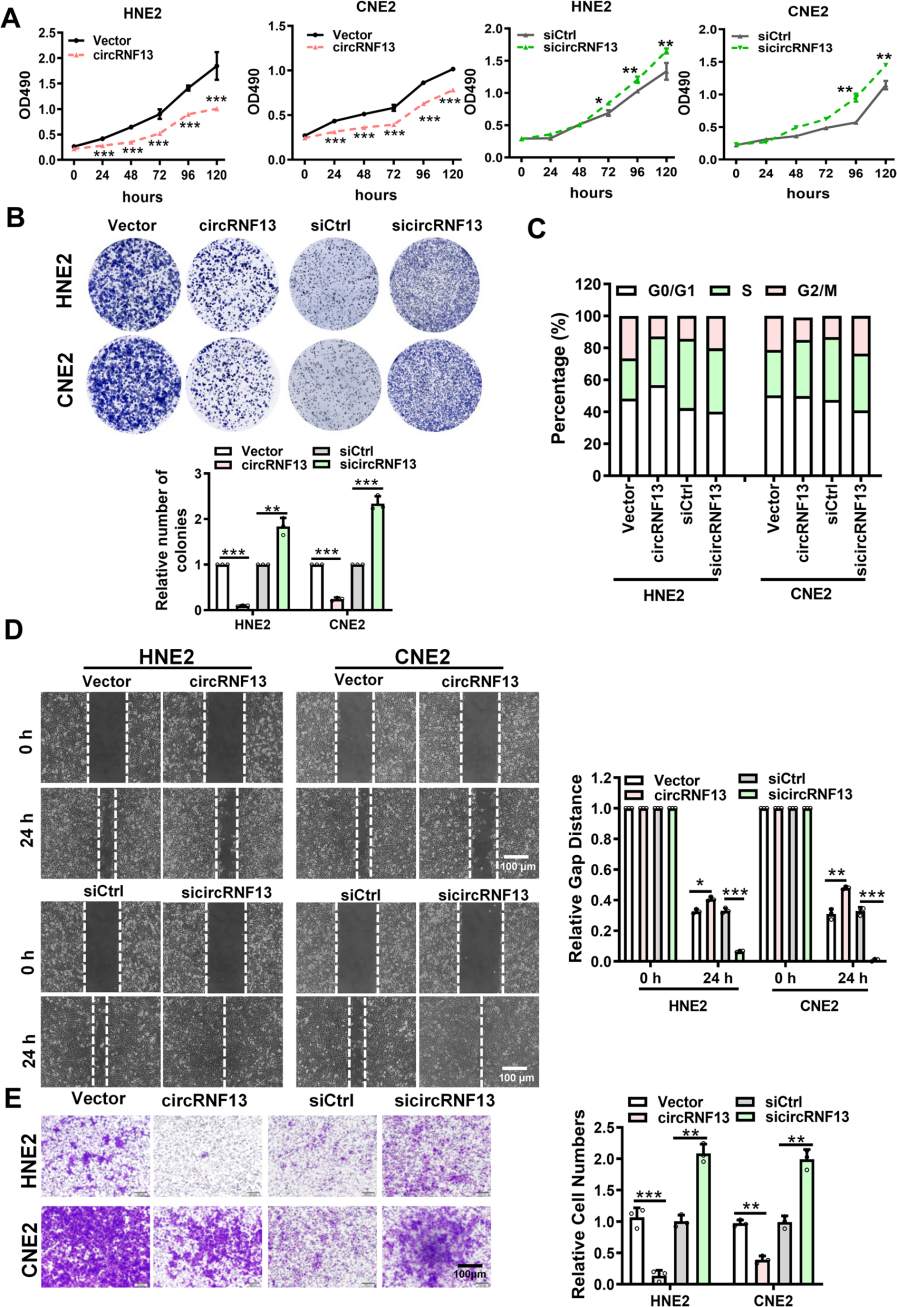
*CircRNF13* inhibits the proliferation, migration, and invasion of NPC. **A** Overexpression of *circRNF13* inhibited, while knockdown of *circRNF13* promoted proliferation of HNE2 and CNE2 cells, as assessed using MTT assay. Data have been represented as mean ± SD. *, *p* < 0.05; **, *p* < 0.05; ***, *p* < 0.001. **B** Clone formation assay showed that overexpression of *circRNF13* inhibited the colony formation capability of HNE2 and CNE2 cells. Data have been represented as mean ± SD. ***, *p* < 0.001. **C ***CircRNF13*’s function in the tumor cell cycle was assessed using flow cytometry. HNE2 and CNE2 cells were stained with PI, after overexpression or knockdown of *circRNF13*. **D ***CircRNF13* inhibited the migration ability of HNE2 and CNE2 cells transfected with sicircRNF13 or *circRNF13* overexpression vector, as assessed using wound-healing assay. Images were acquired at 0 and 24 h. Data have been represented as mean ± SD. *, *p* < 0.05; ***, *p* < 0.001. **E ***CircRNF13* inhibited the invasive ability of HNE2 and CNE2 cells after knockdown or overexpression of *circRNF13*, as assessed using Transwell invasion assays. Images were acquired at 24 h, and are representative of three independent experiments. Data have been represented as mean ± SD. **, *p* < 0.01; ***, *p* < 0.001

To explore the function of *circRNF13* in NPC, a *circRNF13* overexpression vector was constructed, and a *circRNF13* siRNA was designed based on the splice site of *circRNF13*. RT-PCR results showed that *circRNF13* overexpression vector or *circRNF13* siRNA successfully overexpressed or knocked down *circRNF13* in NPC cells and had no effect on linear RNF13 mRNA (Fig. S2). MTT and colony formation assays showed that overexpression of *circRNF13* inhibited NPC cell proliferation, and knockdown of *circRNF13* promoted NPC cell proliferation (Fig. 2A, B). Flow cytometry results also showed that cells were stalled in the G1 phase upon overexpression of *circRNF13*, while knockdown of *circRNF13* increased the number of cells in the G2/M phase (Fig. 2C). Results of the wound-healing experiments showed that the migration capacity of NPC cells was significantly reduced in response to high *circRNF13* expression and induced in response to low *circRNF13* expression (Fig. 2D). Transwell assays showed that overexpression of *circRNF13* inhibited NPC cell invasion and knockdown of *circRNF13* promoted invasion (Fig. 2E). Based on these observations, we concluded that *circRNF13* inhibits NPC cell proliferation, migration, and invasion.

### *CircRNF13* inhibits NPC growth and metastasis in vivo

**Fig. 3:**
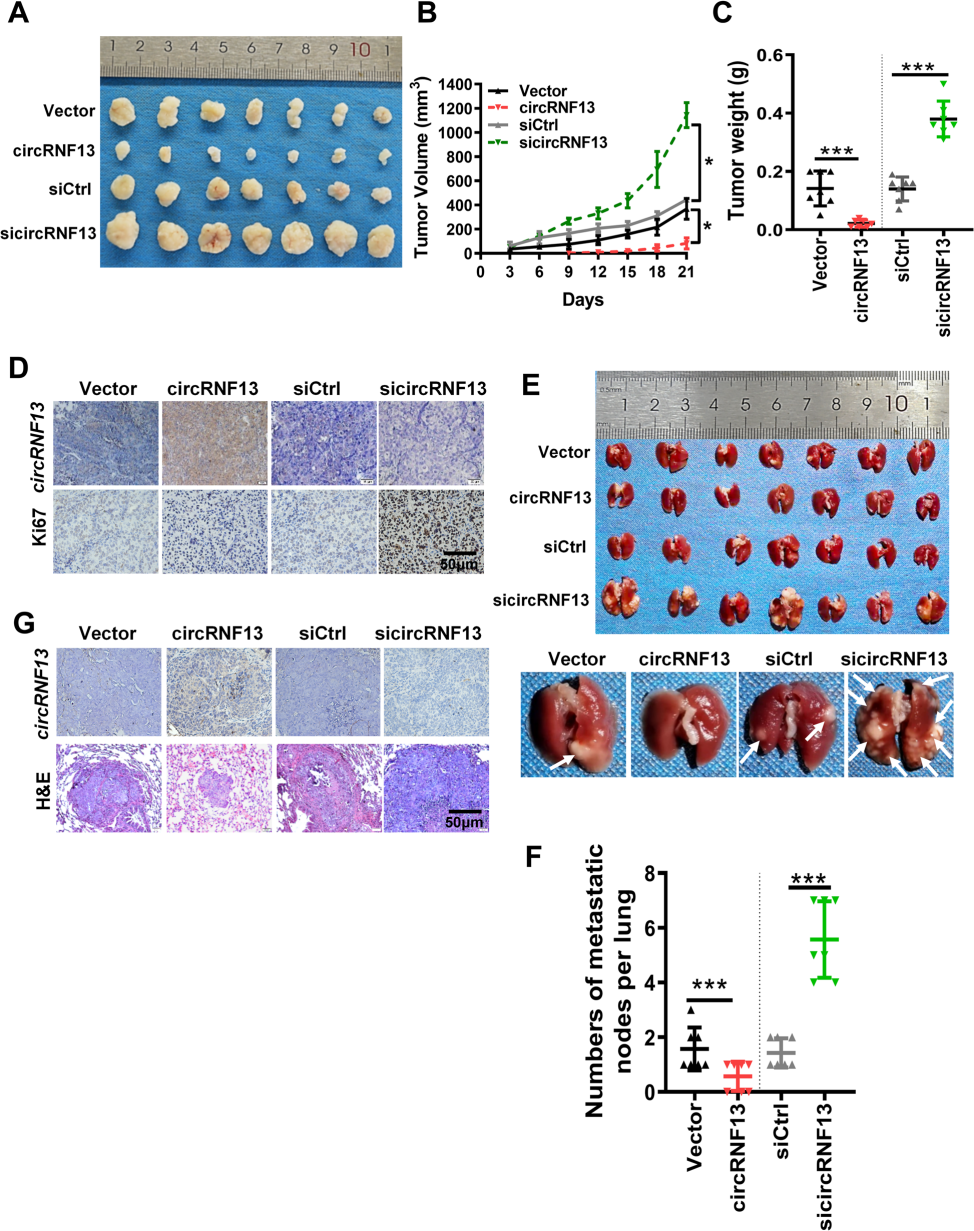
*CircRNF13* inhibits NPC growth and metastasis in vivo. **A** Images of subcutaneous tumor formation in nude mice at 4 weeks. Mice were injected with 2 × 10^6^ CNE2 cells, with knockdown or overexpression of *circRNF13*. **B**, **C** Tumor volumes (B) and tumor weights (C) were measured for each group (*n* = 7 per group). Data have been represented as mean ± SD. *, *p* < 0.05; ***, *p* < 0.001. **D** Representative images of in situ hybridization for *circRNF13* and immunohistochemical staining for Ki-67 expression in subcutaneous tumors (200 × , scale bar: 50 μm). **E** Images of visible nodules on the lung surface. CNE2 cells transfected with scrambled siRNA, empty vector, sicircRNF13, or *circRNF13* overexpression vector were injected into each nude mouse tail vein (*n* = 7 for each group), and the mice were sacrificed 8 weeks later. **F** The number of lung metastatic nodules on each lung surface was quantified. Data have been represented as mean ± SD (each point represents one mouse; *n* = 7 per group; right). ***, *p* < 0.001. **G** Representative images of *circRNF13* expression, as assessed using in situ hybridization, and lung metastatic tumor foci after H&E staining (200 × , scale bar: 50 μm)

Next, we investigated the effect of *circRNF13* on NPC cell proliferation and metastasis in vivo. CNE2 cells transfected with sicircRNF13 or *circRNF13* overexpression vector were inoculated into female nude mice, to establish a xenograft model, by means of subcutaneous or tail vein injection, respectively. The results of subcutaneous tumorigenesis experiments showed that the *circRNF13*-overexpressing nude mice exhibited smaller tumors, while the *circRNF13* knockdown group presented larger subcutaneous tumors than the control group (Fig. 3A-C). Paraffin embedding, sectioning, and immunohistochemical staining showed that Ki-67 expression in the *circRNF13* overexpression group was significantly lower than that in the *circRNF13* knockdown group (Fig. 3D). The results of the tail vein lung metastasis assay showed that the number of lung nodules in the *circRNF13* overexpression group was significantly lower than that in the control group, while there was an increase in the number of lung nodules in the *circRNF13* knockdown group (Fig. 3E-F). H&E staining of lung nodules also revealed that the number of NPC lung metastases in the *circRNF13* overexpression group was significantly lower than that in the *circRNF13* knockdown group (Fig. 3G). Taken together, the above results suggested that *circRNF13* inhibits the proliferation and metastasis of NPC in vitro and in vivo.

### *CircRNF13* may inhibit migration and invasion of NPC by inhibiting glycolysis

To investigate the molecular mechanism of *circRNF13* on NPC proliferation and metastasis, we performed liquid chromatography-tandem mass spectrometry (LC–MS/MS) in CNE2 cells transfected with sicircRNF13 or scrambled siRNA. The score and area values of the LC–MS/MS data were selected as the evaluation criteria to compare proteins’ expression between the two groups. A total of 294 differentially expressed proteins were identified (Table S5). Enrichment analysis by IPA (Ingenuity Pathway Analysis) software showed that many molecules in the metabolic-related pathways, especially the glycolysis pathway, were included (Fig. S3A-B). These results imply that the glycolysis pathway might be regulated by *circRNF13* in NPC.

**Fig. 4:**
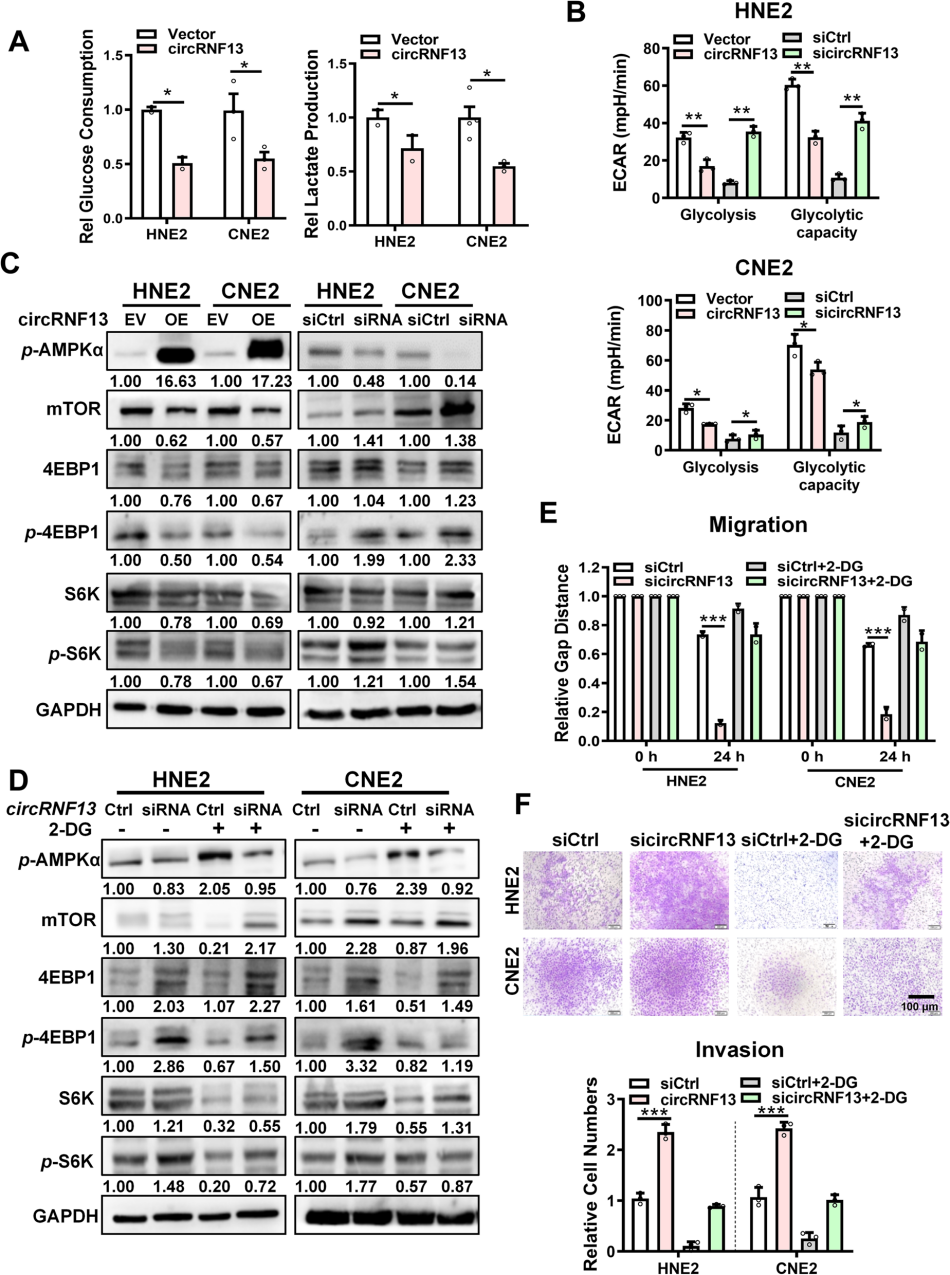
*CircRNF13* inhibits glycolysis in NPC cells. **A** Glucose consumption and lactate production in HNE2 and CNE2 cells were examined using an automated biochemical analyzer, upon *circRNF13* overexpression. Data have been represented as mean ± SD from three independent experiments. *, *p* < 0.05. **B** The extracellular acidification rate (ECAR) in HNE2 and CNE2 cells was measured using Seahorse XF assay, in response to *circRNF13* overexpression or knockdown. Glycolysis, glycolytic capacity, and glycolytic reserve were analyzed. Data have been represented as mean ± SD. *, *p* < 0.05; **, *p* < 0.01. **C** Expression levels of downstream targets of phosphorylated AMPKα and mTOR, S6K and 4EBP1, were measured using western blotting in HNE2 and CNE2 cells in response to *circRNF13* overexpression or knockdown. **D** Expression levels of downstream targets of phosphorylated AMPKα and mTOR, S6K and 4EBP1, were measured using western blotting in HNE2 and CNE2 cells treated with the glycolysis inhibitor 2-DG, in response to *circRNF13* overexpression or knockdown. **E** The glycolysis inhibitor 2-DG attenuated the effect of *circRNF13* on NPC cell migration in HNE2 and CNE2 cells transfected with sicircRNF13, as assessed using wound-healing assay. All experiments were repeated at least three times. Data have been represented as mean ± SD. ***, *p* < 0.001. **F** The glycolysis inhibitor 2-DG weakened the function of *circRNF13* on NPC cell invasion when HNE2 and CNE2 cells transfected with sicircRNF13 were treated with the glycolysis inhibitor 2-DG, as assessed using Transwell assays. All experiments were repeated at least three times. Data have been represented as mean ± SD. ***, *p* < 0.001

To explore the function of *circRNF13* in the glycolysis pathway, biochemical assays were performed, which revealed that overexpression of *circRNF13* inhibited glucose uptake and lactate production in NPC cells (Fig. 4A). Seahorse assay revealed that overexpression of *circRNF13* caused a significant decrease in the glycolytic capacity of CNE2 and HNE2 cells, while knockdown of *circRNF13* significantly increased glycolysis (Fig. 4B, Fig. S3C).

The glycolysis pathway can be monitored by measuring the intracellular AMPK levels. When the glycolysis process is inhibited, intracellular AMPK levels are activated by phosphorylation, and activated AMPK inhibits protein synthesis and translation by inhibiting mTOR, which in turn inhibits tumor growth and metastasis [16, 17]. To further clarify whether cricRNF13 inhibits the proliferation and migration of NPC cells by inhibiting glycolysis, the intracellular phosphorylation of AMPK and mTOR was examined in HNE2 and CNE2 cells. Western blotting revealed that overexpression of *circRNF13* in NPC cells significantly upregulated phosphorylated AMPKα and downregulated mTOR, further downregulating the expression of downstream targets of mTOR, pS6K and 4EBP1. Knockdown of *circRNF13*, on the other hand, induced the opposite effect (Fig. 4C). The glycolysis inhibitor 2-DG was used to block the glycolysis pathway. When the glycolysis inhibitor 2-DG was added, knockdown of *circRNF13* did not downregulate phosphorylated AMPKα (Fig. 4D). This data revealed that knockdown of *circRNF13* promoted NPC cell migration and invasion, and this effect was diminished upon addition of 2-DG (Fig. 4E-F, Fig. S3D). These results suggested that *circRNF13* may inhibit the migration and invasion of NPC by inhibiting glycolysis.

### *CircRNF13* inhibits glycolysis in NPC cells by promoting GLUT1 ubiquitination

**Fig. 5:**
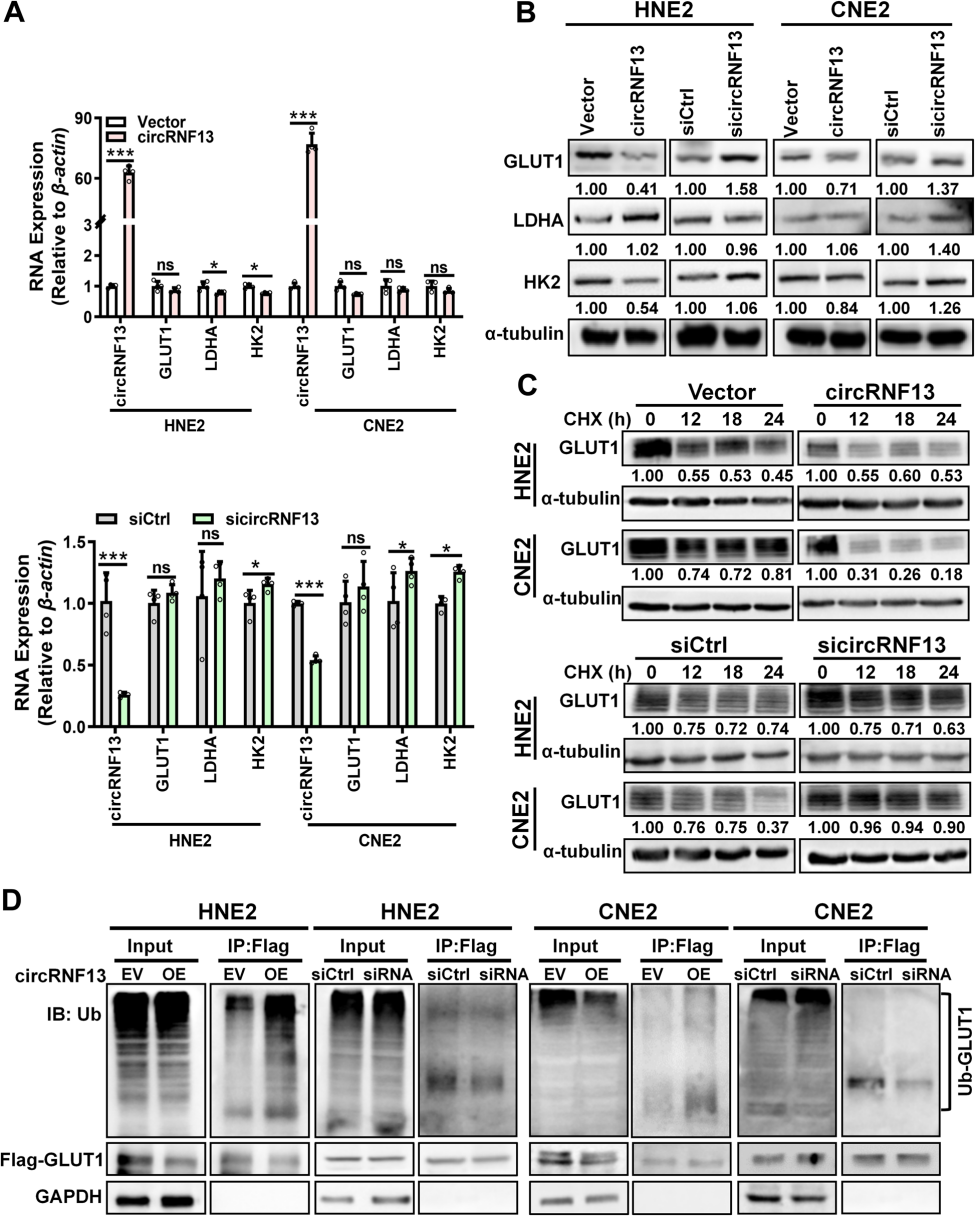
*CircRNF13* inhibits glycolysis in NPC cells by promoting GLUT1 ubiquitination. **A** The mRNA levels of GLUT1, HK2, and LDHA in HNE2 and CNE2 cells were examined using RT-PCR, upon overexpression and knockdown of *circRNF13*. Data have been represented as mean ± SD; ns, no significance; ***, *p* < 0.001. **B** The expression levels of GLUT1, HK2, and LDHA proteins were examined using western blotting in HNE2 and CNE2 cells, upon overexpression and knockdown of *circRNF13*. **C** Degradation of GLUT1 was detected in HNE2 and CNE2 cells using western blotting, after overexpression and knockdown of *circRNF13* and treatment with 50 μg/mL cycloheximide (CHX). **D** Ubiquitination levels of GLUT1 protein were detected in HNE2 and CNE2 cells using pull-down with an anti-Flag antibody and western blot with an anti-ubiquitin antibody, after overexpression or knockdown of *circRNF13*

To investigate the molecular mechanisms by which *circRNF13* regulates the glycolytic process in NPC cells, the primary molecules of the glycolysis pathway, including GLUT1, LDHA, and HK2, were examined using RT-PCR and western blotting experiments in HNE2 and CNE2 cells. The data revealed that GLUT1 protein levels were significantly reduced in response to overexpression of *circRNF13* and significantly increased upon knockdown of *circRNF13*, while the mRNA expression levels were not significantly altered. *CircRNF13* had a weak effect on the expression of LDHA and HK2, at both the mRNA and protein levels (Fig. 5A, B).These data imply that *circRNF13* may regulate glycolysis primarily through the modulation of GLUT1.

We speculated whether *circRNF13* affected glycolysis by influencing the protein stability of GLUT1, so CNE2 and HNE2 cells were treated with cycloheximide (CHX) after overexpression or knockdown of *circRNF13*. Western blotting showed that the stability of GLUT1 decreased in response to overexpression of *circRNF13*, while it increased upon knockdown of *circRNF13* (Fig. 5C). Overexpression of *circRNF13* promoted GLUT1 ubiquitination, whereas knockdown of *circRNF13* decreased protein ubiquitination levels of GLUT1, as demonstrated using an anti-ubiquitin antibody (Fig. 5D). These results suggested that *circRNF13* may inhibit glycolysis in NPC cells by regulating ubiquitin-mediated degradation of GLUT1.

### *CircRNF13* promotes the stability of SUMO2 by directly binding to the 3′-UTR of SUMO2 mRNA

To investigate how *circRNF13* regulates GLUT1 ubiquitination, the LC–MS/MS data were reanalyzed, upon which SUMO2 caught our attention (Table S6). SUMO2 is a member of the SUMO family. SUMOylation is a type of ubiquitin-like protein modification that regulates protein degradation via the ubiquitination pathway. To explore the function of SUMO2 in NPC cells, SUMO2 was overexpressed in NPC cells; the overexpression was confirmed using RT-PCR and western blotting (Fig. S4A). The MTT assay showed that SUMO2 promoted NPC cell proliferation (Fig. S4B). Wound-healing and Transwell assays also demonstrated that SUMO2 induced NPC cell migration and invasion (Fig. S4C-D). Seahorse assays showed that SUMO2 inhibited glycolysis in NPC (Fig. S4E). Therefore, these data indicated that SUMO2 inhibits glycolysis in NPC.

**Fig. 6:**
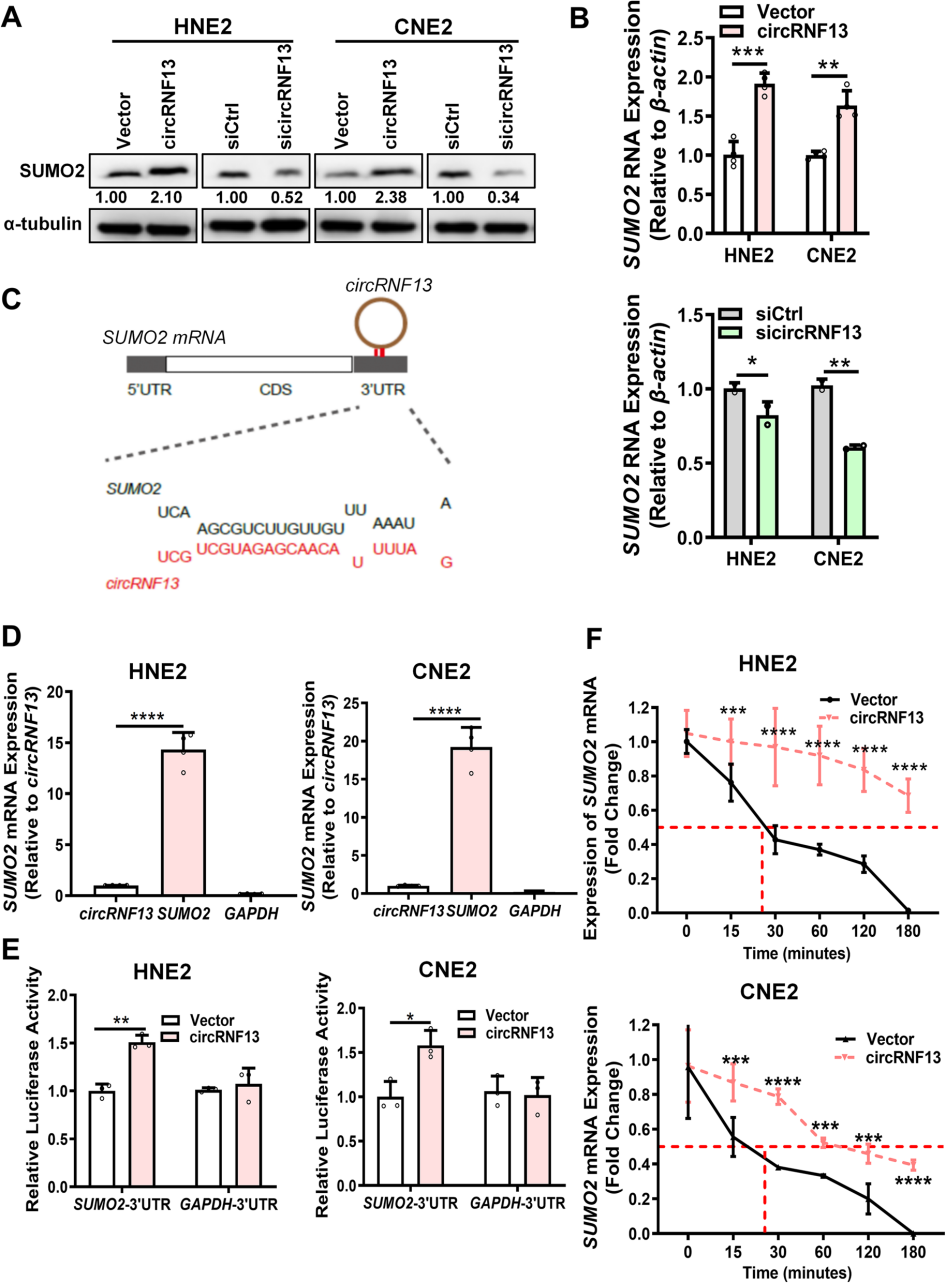
*CircRNF13* directly binds to and stabilizes SUMO2 mRNA, to upregulate its expression **A.** Expression of SUMO2 protein was examined using western blotting in HNE2 and CNE2 cells, after overexpression and knockdown of *circRNF13*. **B** Expression of SUMO2 mRNA was examined in HNE2 and CNE2 cells using RT-PCR, after overexpression and knockdown of *circRNF13*. **C** Bioinformatics prediction using the RNAhybrid website revealed that *circRNF13* has a continuous binding site with the 3′-UTR of SUMO2 mRNA. **D ***circRNF13* was found to bind to the 3′-UTR of SUMO2 mRNA in HNE2 and CNE2 cells using RNA pull-down assay. GADPH was used as a negative control. Data have been represented as mean ± SD. ***, *p* < 0.001. **E***circRNF13* enhanced the luciferase activity of the SUMO2 mRNA 3′-UTR in HNE2 and CNE2 cells, as assessed using dual luciferase reporter assay. The 3′-UTR of GADPH was used as a negative control. Data have been represented as mean ± SD. *, *p* < 0.05; **, *p* < 0.01. **F.** Degradation of SUMO2 was detected in HNE2 and CNE2 cells using RT-PCR, after overexpression and knockdown of *circRNF13* and treatment with 2 μg/mL actinomycin D. Data have been represented as mean ± SD. ***, *p* < 0.001, ****, *p* < 0.0001

To identify whether *circRNF13* interacts with SUMO2 to regulate proliferation, migration, and invasion, western blotting and RT-PCR were performed, which revealed that overexpression of *circRNF13* significantly induced the expression of SUMO2 at both the RNA and protein levels. The opposite results were obtained upon knockdown of *circRNF13* (Fig. 6A, B). Bioinformatics analysis revealed the presence of a continuous binding motif between *circRNF13* and the 3′-UTR of SUMO2 (Fig. 6C). RNA pull-down assays demonstrated that biotin-labeled *circRNF13* could pull-down SUMO2 mRNA in NPC cells (Fig. 6D). Dual-luciferase experiments demonstrated that overexpression of *circRNF13* increased the fluorescence activity of the SUMO2 3′-UTR in CNE2 cells (Fig. 6E). Actinomycin D (2 μg/mL) was used to measure whether *circRNF13* regulates the stability of SUMO2 by binding to its 3′-UTR in HNE2 and CNE2 cells, upon overexpression of *circRNF13*. Compared to the control group, the stability of SUMO2 mRNA was significantly increased in the *circRNF13* overexpression group (Fig. 6F). These data indicated that *circRNF13* regulates SUMO2 expression by binding to its 3′-UTR.

### *CircRNF13* promotes ubiquitination of GLUT1 via SUMO2

**Fig. 7:**
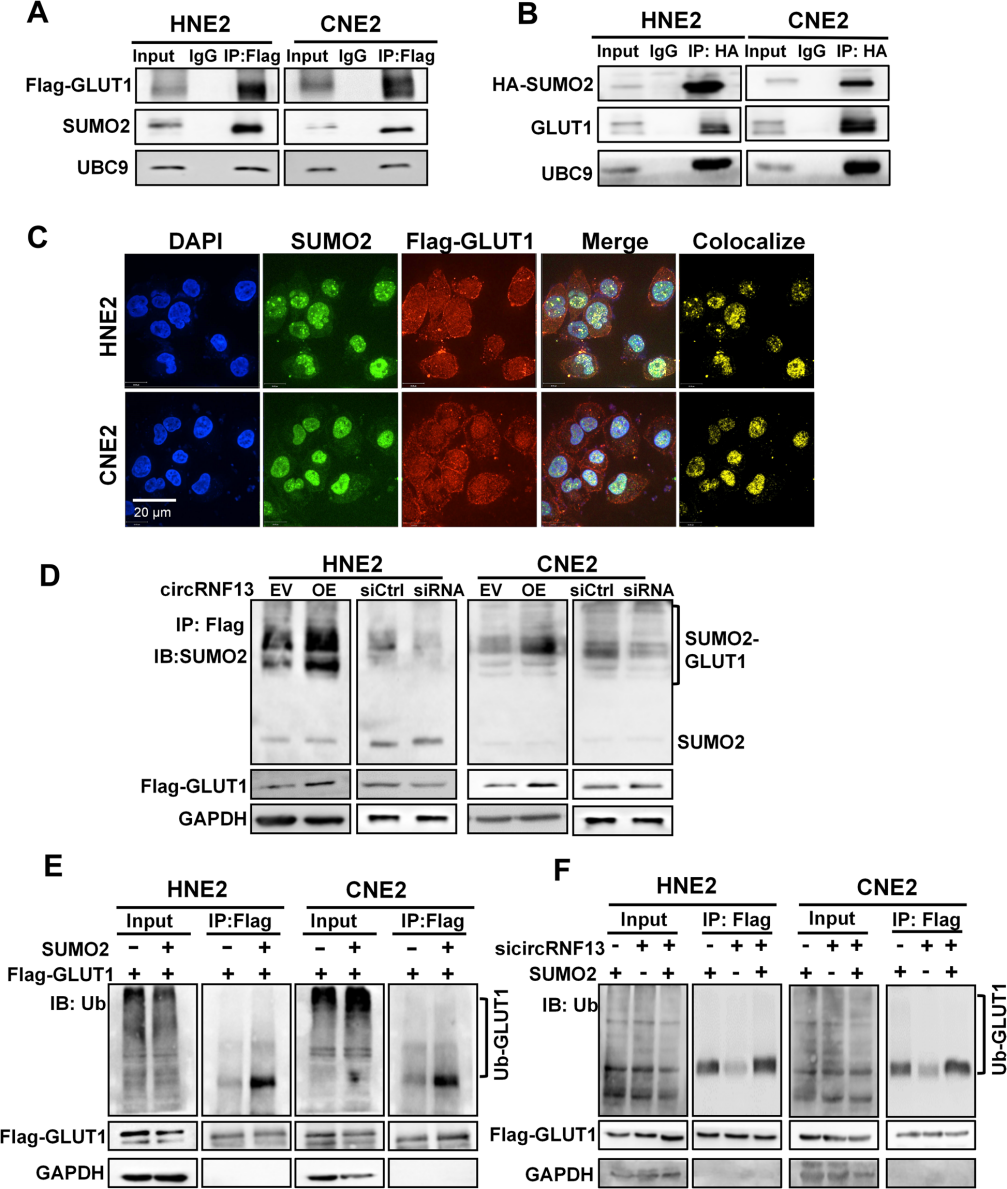
*CircRNF13* promotes ubiquitination of GLUT1 via SUMO2. **A** The interaction between GLUT1, SUMO2, and UBC9 was examined using immunoprecipitation and an anti-Flag (GLUT1) antibody in HNE2 and CNE2 cells transfected with Flag-GLUT1 vector, followed by western blotting using the anti-SUMO2 and anti-UBC9 antibodies. **B** The interaction between SUMO2, GLUT1, and UBC9 proteins was examined using immunoprecipitation with an anti-HA (SUMO2) antibody in HNE2 and CNE2 cells transfected with HA-SUMO2 vector, followed by western blotting using anti-GLUT1 and anti-UBC9 antibodies. **C** Immunofluorescence experiments performed using anti-Flag (GLUT1) and anti-SUMO2 antibodies in HNE2 and CNE2 cells showed that GLUT1 and SUMO2 were co-localized. DAPI-stained nucleus: blue; anti-Flag (GLUT1): red; anti-SUMO2: green; co-localization of GLUT1 and SUMO2: yellow; the merged image represents the overlap of DAPI, GLUT1, and SUMO2 (scale bar: 20 μm). **D** Immunoprecipitation experiments using an anti-Flag-GLUT1 antibody, followed by western blotting using an anti-SUMO2 antibody, were performed, to identify the SUMOylation level of the GLUT1 protein in HNE2 and CNE2 cells transfected with sicircRNF13 or *circRNF13* overexpression vector. **E** The ubiquitination level of GLUT1 protein was detected in HNE2 and CNE2 cells using pull-down with an anti-Flag-GLUT1 antibody, followed by western blotting with an anti-ubiquitin antibody, to determine whether SUMO2 affects GLUT1 ubiquitination. **F** A pull-down experiment using an anti-Flag-GLUT1 antibody, followed by western blot using an anti-ubiquitin antibody, were performed to identify the ubiquitination level of GLUT1 protein in HNE2 and CNE2 cells co-transfected with sicircRNF13 and SUMO2 overexpression vector

To investigate whether *circRNF13* regulates GLUT1 ubiquitination by binding to SUMO2, bioinformatics analysis was performed and revealed that there were two binding motifs of SUMO2 protein on the GLUT1 protein (Fig. S5A). Co-immunoprecipitation (Co-IP) experiments were performed to confirm the interaction between GLUT1 and the SUMO2 protein using an anti-Flag-GLUT1 antibody. During SUMOylation, UBC9 is the only ligase that can directly recognize the substrate and catalyze the binding of the C-terminal carboxyl group of the SUMO protein to the substrate protein for modification. Our Co-IP experiment also revealed an interaction between GLUT1 and UBC9 (Fig. 7A,B). Immunofluorescence also revealed co-localization of the SUMO2 and GLUT1 proteins (Fig. 7C). To understand the molecular basis of SUMO2 in NPC, SUMOylation of GLUT1 was examined in HNE2 and CNE2 cells, upon overexpression or knockdown of *circRNF13*. Western blotting showed that overexpression of *circRNF13* promoted SUMOylation of GLUT1, and knockdown of *circRNF13* inhibited SUMOylation of GLUT1 (Fig. 7D).

To determine whether SUMO2 participates in the ubiquitination of GLUT1 regulated by *circRNF13*, SUMO2 was overexpressed in NPC cells; western blotting showed that overexpression of SUMO2 inhibited GLUT1 expression (Fig. S5B). Overexpression of SUMO2 induced GLUT1 degradation in HNE2 and CNE2 cells treated with cycloheximide (CHX) (Fig. S5C). Ubiquitination experiments demonstrated that overexpression of SUMO2 accelerated the ubiquitination of GLUT1 (Fig. 7E). Knockdown of *circRNF13* weakened the SUMO2-induced ubiquitination level of GLUT1 in HNE2 and CNE2 cells co-transfected with sicircRNF13 and SUMO2 overexpression vector (Fig. 7F). These results suggested that *circRNF13* promotes the ubiquitination of GLUT1, to inhibit GLUT1 expression, thus suppressing the glycolytic process in NPC cells. These data suggested that *circRNF13* regulates GLUT1 ubiquitination by binding to SUMO2.

### *CircRNF13* inhibits proliferation, migration, and invasion of NPC cells via SUMO2

**Fig. 8:**
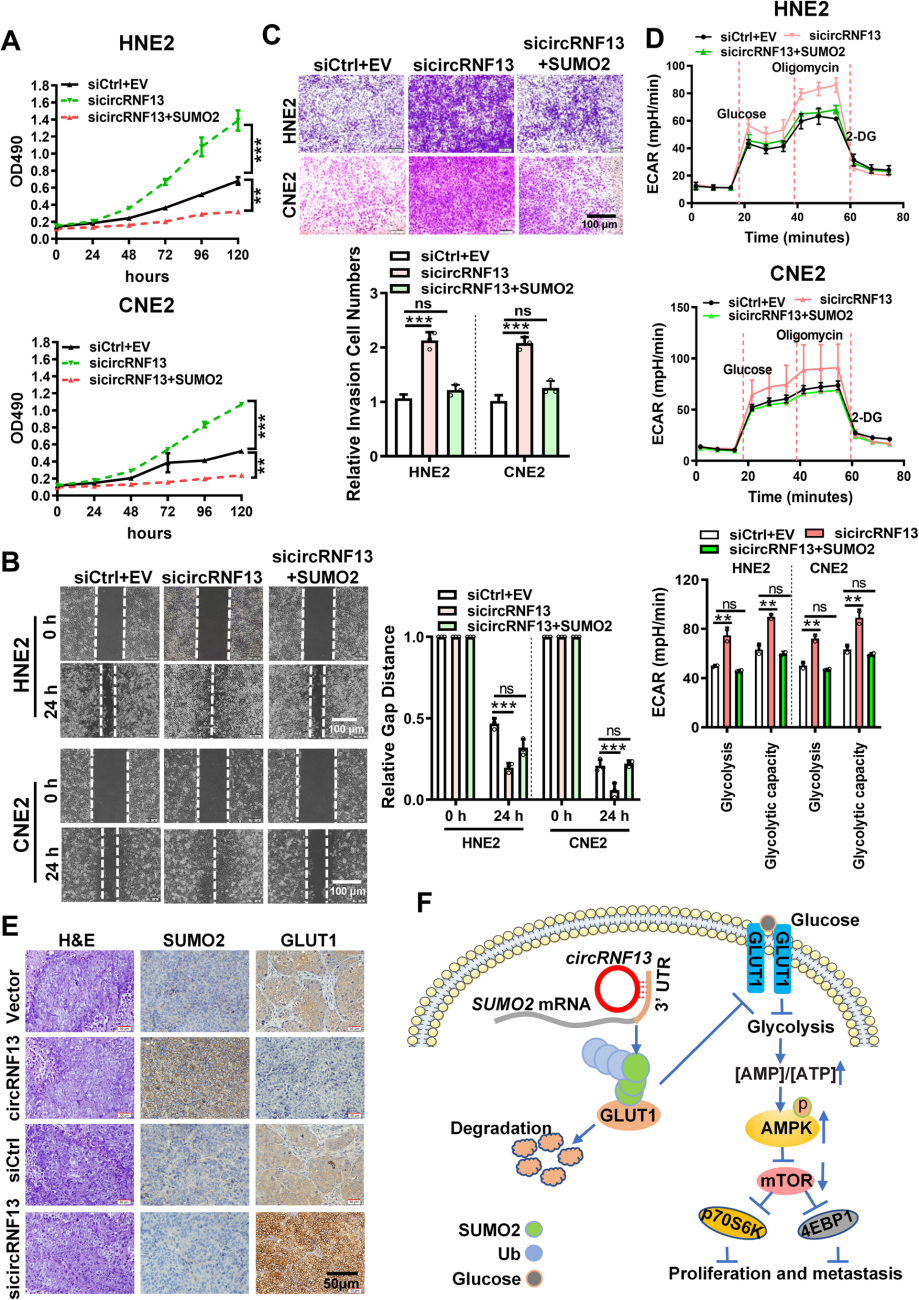
*CircRNF13* inhibits the proliferation and metastasis of NPC via SUMO2. **A** Overexpression of SUMO2 rescued the *circRNF13* knockdown-mediated altered proliferative capacity of HNE2 and CNE2 cells, as assessed using MTT assay. Data have been represented as mean ± SD. **, *p* < 0.01; ***, *p* < 0.001. **B** Overexpression of SUMO2 decreased the *circRNF13* knockdown-mediated altered migration of HNE2 and CNE2 cells, as assessed using wound-healing assays. All experiments were repeated at least three times (Scale bar: 100 μm). Data have been represented as mean ± SD; ns, no significance; ***, *p* < 0.001. **C.** Overexpression of SUMO2 reduced the *circRNF13* knockdown-mediated altered invasive ability of HNE2 and CNE2 cells, as assessed using Transwell assays. All experiments were repeated at least three times (Scale bar: 100 μm). Data have been represented as mean ± SD; ns, no significance; ***, *p* < 0.001. **D** The extracellular acidification rate (ECAR) was measured using Seahorse XF assay in HNE2 and CNE2 cells co-transfected with sicircRNF13 and SUMO2 overexpression vector. Glycolysis, glycolytic capacity, and glycolytic reserve were analyzed. All experiments were repeated at least three times. Data have been represented as mean ± SD; ns, not significant; **, *p* < 0.01. **E** Representative images of SUMO2 and GLUT1 expression in lung tissues of nude mice, as determined using immunohistochemistry (*n* = 7 per group, 200 × , scale bar: 50 μm). **F** Schematic diagram of the molecular mechanism of *circRNF13* in NPC. *circRNF13* may activate and stabilize the SUMO2 protein by binding to the 3′-UTR of SUMO2 mRNA. Upregulation of SUMO2 promotes GLUT1 degradation through SUMOylation and ubiquitination of GLUT1, which regulates the AMPK pathway by inhibiting glycolysis, ultimately resulting in the proliferation and metastasis of NPC

MTT assays were performed to determine whether *circRNF13* inhibits NPC cell proliferation, migration, and invasion via SUMO2, which revealed that overexpression of SUMO2 rescued the *circRNF13* knockdown-mediated altered proliferation ability of NPC cells (Fig. 8A). Wound-healing and Transwell assays showed that SUMO2 rescued the *circRNF13* knockdown-mediated altered migration and invasive abilities of NPC cells (Fig. 8B, C). Seahorse assay also showed that overexpression of SUMO2 rescued the altered glycolytic stress in NPC cells caused by knockdown of *circRNF13* (Fig. 8D).

Immunohistochemistry for SUMO2 and GLUT1 was also performed in mouse tissues, which showed that SUMO2 was expressed at low levels, while GLUT1 was highly expressed in subcutaneous tumor sections of nude mice in the *circRNF13* overexpression group (Fig. 8E). These results underscored the significance of *circRNF13* in NPC proliferation and metastasis. *circRNF13*, acting as a tumor suppressor, directly binds to the 3′-UTR of SUMO2 and prolongs the half-life of SUMO2 mRNA. Upregulation of SUMO2 promotes GLUT1 degradation through SUMOylation and ubiquitination of GLUT1, which regulates the AMPK-mTOR pathway by inhibiting glycolysis, ultimately resulting in proliferation and metastasis of NPC (Fig. 8F).

## Discussion

In this study, we identified a new circRNA, *circRNF13*, which is expressed at low levels in NPC and regulates glycolysis in NPC cells by binding to the 3′-UTR of SUMO2 mRNA, to enhance GLUT1 degradation, resulting in the proliferation and metastasis of NPC. We screened the circRNAs from our RNA-seq data on the NPC cell line 5-8F. From the RNA-seq data, some new circRNAs were identified, which have not been reported yet. Some of the other identified circRNAs have already been reported to play important functions in the development of NPC. For example, *circMAN1A2* is highly expressed in the serum of patients, including NPC patients, and can be used as a potential molecular marker for tumors [24].

In our study, we only selected *circRNF13*, because of its high abundance in NPC cells. However, it was more highly expressed in NPE tissues than in NPC tissues. In NPC, some circRNAs have been reported to be oncogenes that promote the development of NPC. Our previous data showed that *circSETD3*, acting as a competing endogenous RNA, promotes NPC migration and invasion by completely binding to *miR-615-5p* and *miR-1538* with MAPRE1 [25]. *CircCRIM1* promotes NPC metastasis and chemoresistance by upregulating FOXQ1 expression through adsorption of *miR-422a* [26]. Wang et al*.* found that *circTGFBR2* acted as a sponge for *miR-107*, to upregulate TGFBR2, resulting in the inhibition of NPC progression [27]. Chen et al*.* found that *circHIPK3* promoted NPC proliferation and invasion by eliminating *miR-4288*-induced inhibition of ELF3 [28]. Fan et al*.* found that *circARHGAP12* promoted NPC cell invasion and metastasis by binding directly to the 3′-UTR of EZR mRNA and promoting its stability [29]. These results suggested that circRNAs play an important role in promoting the development of NPC. In this study, we found that *circRNF13*, acting as a tumor suppressor, directly binds to and stabilizes SUMO2 mRNA, to promote SUMO2 protein expression, thereby inhibiting the proliferation and metastasis of NPC. Thus, it can serve as a new model for research on the mechanism of circRNA regulation in tumor development and for the development of drug targets.

Metabolic reprogramming and invasive metastasis are important features of tumors that have long been the focus of attention. Recent studies have revealed that these two malignant phenotypes are not independent, but are actually closely linked. For example, the transmembrane glycoprotein CD147 promotes MCT1 expression, leading to increased lactate secretion, which activates the PI3K/Akt/MDM2 pathway, increasing p53 degradation and promoting the proliferation and metastasis of hepatocellular carcinoma cells [30, 31]. PGC1-α, a core regulator of metabolism, is significantly overexpressed in cancer and leads to a significant increase in tumor invasion and metastasis [32]. Morrison et al*.* found that melanomas with high expression of the monocarboxylate transporter MCT1 could resist oxidative stress by taking up lactic acid in the circulatory system, resulting in a greater metastatic capacity [33]. In conclusion, abnormal metabolic function of tumor cells can enhance tumor invasion and metastasis through multiple pathways. In this study, we found that *circRNF13* inhibited glycolysis in NPC cells by suppressing the expression of GLUT1, which inhibited proliferation and metastasis. GLUT1 is an important protein in the process of glycolysis, and malignant tumor cells characteristically overexpress GLUT1, which provides favorable conditions for glycolysis through massive glucose uptake, thus providing more energy and synthetic raw materials for tumor cells. In addition, the large amount of lactic acid produced by glycolysis alters the microenvironment of tumor cells, which is more favorable for tumor cell invasion and metastasis. Designing *circRNF13*-based inhibitors against GLUT1, to block tumor cell metabolism, is also a promising strategy for future malignancy treatment.

GLUT1 is the most widely distributed glucose transporter protein [34]. High expression of GLUT1 has been reported in various tumors, such as liver, gastric, and breast cancers [35–38]. While the transcriptional regulation of GLUT1 has been well studied, the post-translational modifications of GLUT1 have relatively few reports, especially ubiquitination modifications of GLUT1. Xu et al*.* found that SALL4 recruits the E3 ubiquitin ligase CUL4B to GLUT1, which reduces the expression levels of GLUT1, and subsequently, inhibits glycolysis in cancer cells [39]. Lin et al*.* found that the E3 ubiquitin ligase family SCF complex Skp2 reduces GLUT1 expression and inhibits glycolysis in tumor cells by ubiquitinating Akt [40]. In this study, we found that SUMO2 directly promotes the degradation of GLUT1 by means of SUMOylation and ubiquitination. This further increases the understanding of the regulatory mechanism of GLUT1. More importantly, blocking the nutritional source of cells by targeting GLUT1 is an important strategy for the development of anti-tumor drugs. Study of GLUT1 ubiquitination and SUMOylation will likely provide new ideas for drug development.

RNF13 is an E3 ubiquitin-protein ligase with a structural domain of a typical RING zinc-finger protein. In this study, we determined that *circRNF13* is a circular splicing of exons 2–8 of the RNF13 gene. Our study demonstrated that *circRNF13* is expressed at low levels in NPC and inhibits its proliferation and metastasis. The expression and function of *circRNF13* are inconsistent with those of its parental gene RNF13, as the latter exerts an oncogenic function. Overexpression of the RNF13 enzyme is apparent in various human cancers, including basal cell carcinoma, melanoma, and ovarian carcinoma [41, 42]. RNF13, which acts as a ubiquitin ligase, participates in cancer invasion and metastasis. In this study, we analyzed the expression of RNF13 in the online GEO NPC database and our clinical NPC samples. There was no difference in RNF13 expression between the NPE and NPC clinical samples. Knockdown of RNF13 also had no effect on the ubiquitination of GLUT1. RNF13 mRNA could not bind to SUMO2 mRNA in the RNA pull-down experiment (data not shown). These results suggested that the function of *circRNF13* is independent of RNF13 mRNA, in promoting ubiquitination and SUMOylation of GLUT1, by binding to SUMO2 mRNA.

## Conclusions

Our results revealed that the novel *circRNF13* plays an important role in the development of NPC through the *circRNF13*-SUMO2-GLUT1 axis. This study implies that *circRNF13* mediates glycolysis in NPC by binding to SUMO2 and provides an important theoretical basis for further elucidating the pathogenesis of NPC and targeted therapy.

## Supplementary Information

**Additional file 1:Suppemental Table 1.** Probes for fluorescence in situ hybridization and RNA pulldown, siRNAs, primers for RT-PCR and vectors. **Suppemental Table 2.** List of antibodies for immunohistochemistry, western blotting, immunofluorescence, and RNA pulldown experiments. **Suppemental Table 3.** The top 20 circRNAs identified in NPC 5-8F cells using the next-generation sequencing of mRNA (RNA-seq). **Suppemental Table 4.** Clinicopathological data of 36 NPC and 12 NPE tissues measured by RT-PCR. **Suppemental Table 5.** Proteomic analysis of the circRNF13-regulated proteins in CNE2 cells transfected with sicircRNF13 or scrambled siRNA identified by the LC-MS/ MS spectrometry. **Suppemental Table 6.** The top 20 of the *circRNF13*-regulated differential proteins in CNE2 cells after knockdown of *circRNF13* identified by the LC-MS/MS spectrometry.

**Additional file 2:Fig. S1.***CircRNF13 *is lowly expression in NPC. A The top 20 circRNAs in the RNA-seq data were tested in NPC (*n* = 36) and NPE (*n* = 12) tissues using RT-PCR. NPE, non-tumor nasopharyngeal epithelial tissues. Data have been represented as mean ± standard deviation (SD). *, *p *< 0.05; **, *p *< 0.01; ****, *p *< 0.0001. B Expression of *circRNF13 *was verified using qPCR in NPC cell lines 5-8F, CNE2, HNE2,HONE1, and 6-10B and normal immortal nasopharyngeal epithelial NP69 cells. **Fig. S2.***CircRNF13 *inhibits proliferation, migration, and invasion of NPC. The overexpression or knockdown efficiencies of *circRNF13 *were measured in HNE2 and CNE2 cells after transfection with *circRNF13 *overexpression vector or *circRNF13 *siRNAs. RNF13 mRNA expression was not affected by transfection. All experiments were repeated at least three times. Data have been represented as mean ± SD. ***, *p *<0.001. **Fig. S3.***CircRNF13 *inhibits glycolysis in NPC cells. A The proteomics profile in CNE2 cells was analyzed upon *circRNF13 *knockdown. A total of 294 differentially expressed proteins between sicircRNF13 and scrambled siRNAs were screened, including 100 proteins upregulated and 194 proteins downregulated by sicircRNF13. B The glycolysis pathway was enriched according to the 294 differential proteins screened from the LC-MS/MS data, using gene set enrichment analysis with the IPA software. C The extracellular acidification rate (ECAR) in HNE2 and CNE2 cells was measured using Seahorse XF assays, in response to circRNF13 overexpression or knockdown. D Representative images of wound-healing assay showed that the glycolysis inhibitor 2-DG attenuated the effect of *circRNF13 *on NPC cell migration when HNE2 and CNE2 cells transfected with sicircRNF13 were treated with it. **Fig. S4.** SUMO2 inhibits proliferation, migration, invasion, and glycolysis in NPC cells. A The overexpression efficiency of SUMO2 was measured in HNE2 and CNE2 cells using RT-PCR and western blotting, after transfection of the SUMO2 overexpression vector. Data have been represented as mean ± SD. ***, *p *<0.001. B Overexpression of SUMO2 inhibited proliferation of HNE2 and CNE2 cells, as assessed using MTT assay. All experiments were repeated at least three times. Data have been represented as mean ± SD. *, *p *< 0.05; **, *p *< 0.01; ***, *p *< 0.001. C The migration ability of HNE2 and CNE2 cells was significantly reduced upon overexpression of SUMO2, as assessed using wound-healing assays. All experiments were repeated at least three times. Data have been represented as mean ± SD. **, *p *< 0.01; ***, *p *< 0.001. D The invasive ability of HNE2 and CNE2 cells was significantly decreased upon overexpression of SUMO2, as assessed using Transwell assays. All experiments were repeated at least three times. Data have been represented as mean ± SD. ***, *p *< 0.001. E The extracellular acidification rate (ECAR) was measured using Seahorse XF assays upon overexpression of SUMO2 in HNE2 and CNE2 cells. Glycolysis, glycolytic capacity, and glycolytic reserve were analyzed. All experiments were repeated at least three times. Data have been represented as mean ± SD. **, *p *< 0.01. **Fig. S5.** SUMO2 promotes degradation of GLUT1. A The binding motifs for SUMOylation on the GLUT1 protein (K245 and K451) were predicted using GPS-SUMO 2.0 website. B Expression of GLUT1 protein was examined in HNE2 and CNE2 cells using western blotting, after overexpression of SUMO2. C Degradation of GLUT1 was detected in HNE2 and CNE2 cells using western blotting, after overexpression of SUMO2 and treatment with 50 μg/mL cycloheximide (CHX).

## Data Availability

All data that support the findings of this study are available from the corresponding authors upon reasonable request.

## Abbreviations

NPC: Nasopharyngeal carcinoma
*circRNA*: Circular RNAs
RNF13: Ring Finger Protein 13
SUMO2: Small Ubiquitin-like Modifier 2
LC–MS/MS: Liquid Chromatography-tandem Mass Spectrometry
IPA: Ingenuity Pathway Analysis
UTR: Untranslated Region
UBC9: Ubiquitin-Conjugating Enzyme 9
GLUT1: Glucose transporter type 1
LDHA: Lactate Dehydrogenase A
HK2: Hexokinase 2
